# Macromolecular Structure of Linearly Arranged Eukaryotic Chromosomes

**DOI:** 10.3390/ijms23169503

**Published:** 2022-08-22

**Authors:** Gaspar Banfalvi

**Affiliations:** Department of Molecular Biotechnology and Microbiology, University of Debrecen, Life Sciences Building 1.102, 1 Egyetem Square, 4010 Debrecen, Hungary; bgaspar@unideb.hu; Tel.: +36-52-512-900 (ext. 62319) or +36-20-580-8325

**Keywords:** nascent DNA, DNA replication, reversible permeabilization, chromosome isolation, forms of interphase chromosomes

## Abstract

Eukaryotic chromosomes have not been visualized during the interphase. The fact that chromosomes cannot be seen during the interphase of the cell cycle does not mean that there are no means to make them visible. This work provides visual evidence that reversible permeabilization of the cell membrane followed by the regeneration of cell membranes allows getting a glimpse behind the nuclear curtain. Reversibly permeable eukaryotic cells have been used to synthesize nascent DNA, analyze the 5′-end of RNA primers, view individual replicons and visualize interphase chromosomes. Dextran T-150 in a slightly hypotonic buffer prevented cells from disruption. Upon reversal of permeabilization, the nucleus could be opened at any time during the interphase. A broad spectrum of a flexible chromatin folding pattern was revealed through a series of transient geometric forms of chromosomes. Linear attachment of chromosomes was visualized in several mammalian and lower eukaryotic cells. The linear connection of chromosomes is maintained throughout the cell cycle showing that rather than individual chromosomes, a linear array of chromosomes is the functional giant macromolecule. This study proves that not only the prokaryotic genome but also linearly attached eukaryotic chromosomes form a giant macromolecular unit.

## 1. Introduction

Upon hypotonic treatment, *E. coli* cells became permeable to carbamoyl phosphate, as judged by assays of ornithine transcarbamoylase [1]. Permeabilization with hypotonic buffer was confirmed in *E. coli* [2]. For the enzymatic synthesis of DNA deoxyribonucleoside triphosphates (dNTPs) were incorporated into permeabilized *Escherichia coli* cells [3]. Replicative DNA synthesis was performed in toluene-treated *E. coli* cells [4] and in *B. subtilis* [5]. DNA synthesis in permeabilized prokaryotic and eukaryotic cells is semiconservative, the products are high-molecular-weight DNA intermediates, and DNA synthesis occurs as extensions of replication sites that remained active in vivo [6,7,8]. Permeable cells played an important role in the clarification of the mechanism of semiconservative replication. The existence of Okazaki fragments with specific tri- and tetranucleotide RNA primers at their 5′ ends was confirmed in the 1980s in *B. subtilis* [7,8,9,10,11]. Non-specific RNA primers in the decanucleotide range were found in murine thymocytes [12].

The connection of chromosomes remained an unsolved problem requiring an explanation as to how individual chromosomes are arranged to secure their stable position in the nucleus. The free ends of chromosomes would suggest their random ligation but would contradict the Rabl orientation known as a classical example of a non-random arrangement of chromosomes [13]. It was Theodor and Marcella Boveri who in 1909 called these distinct domains chromosome territories [14]. The works of Rabl and Boveri suggested that in animal cells interphase chromosomes adopt a form of territorial organization where inter-chromosomal contacts are minimized [15,16]. After the rise, fall and resurrection of chromosome territories it has been generally accepted that chromosomes are distinct domains. Metaphase chromosomes were visualized under a light microscope and identified [15,16,17,18,19].

The FISH technique has been used to visualize the location of chromosomes. It was demonstrated that chromosomes form individual domains [20,21,22]. In diploid cells, homologous chromosomes were reported to be far apart from each other [23]. Haplotype reconstruction using genome-wide chromatin organization supported these findings by demonstrating that chromosome haplotypes in diploid cells do not interact frequently with each other [24], probably because genes are positioned at the surface of chromosome territories [25,26].

The prediction regarding the chromosome arrangement was tested in interphase chromosomes of Chinese hamster cells and supported the Rabl-model [19]. The present study provides visual evidence that condensing interphase and metaphase chromosomes are linearly attached. The predictions of the Rabl-model are supported by the linear arrays of chromosomes that are maintained during the cell cycle serving the stable position of chromosomes in the nucleus.

## 2. Organization and Visualization of the Nuclear Material

The nuclear material of eukaryotic cells, before condensing to chromosomes, is a colorable substance within the eukaryotic nuclei reflected by its name ‘chromatin’ [27]. The nuclei of mammalian cells can be visualized by microscopy, imaging or flow cytometry (of chromatin are preferentially stained with different types of fluorophores such as methylene blue–eosin Y (Giemsa) stain and fluorescent dyes such as ethidium bromide, 4′,6′-diamidino-2-phenylindole (DAPI), UV-excitable blue fluorescent Hoechst 33342, green fluorescent YO-PRO™ dye that can enter apoptotic cells, whereas red-fluorescent propidium iodide (PI) cannot [3].

**Figure 1:**
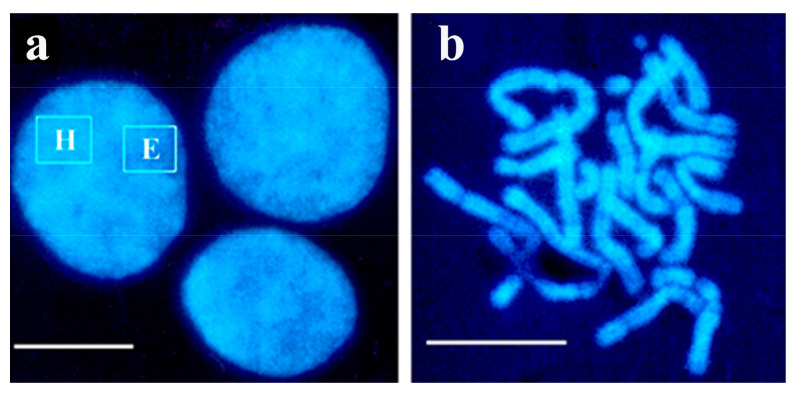
Interphase chromatin (**a**) and metaphase chromosomes (**b**) were isolated from Chinese hamster ovary (CHO) cells. DAPI staining of nuclear structures shows the more condensed heterochromatin (H) (facultative or constitutive) and the less condensed euchromatin (E). Scale bar 5 µm. Reprinted with permission from Ref. [30]. Copyright year 2009, Springer Science + Business Media BV, Dordrecht, Figure 3.4.

Interphase chromosomes are not seen in the nucleus under the microscope. They become visible when the double-layered nuclear membrane is disrupted during cell division revealing the tightly packed chromosomes in metaphase consisting of DNA and histone proteins (https://www.gov/genetics-glossary/Metaphase, accessed on 11 April 2022. Two types of chromatin structures can be seen under the microscope, interphase nuclei (Figure 1a) and metaphase chromosomes (Figure 1b). Chromatin was originally classified into two domains, euchromatin and heterochromatin, based on its density [28]. The definition of the chromatin domains has been refined since, with euchromatin representing the gene-rich, transcriptionally active, hyperacetylated, hypomethylated chromatin. The more condensed heterochromatin is transcriptionally inactive, gene-poor, hypoacetylated and hypermethylated [29]. The two generally known and visible chromatin structures, namely interphase nuclei and metaphase chromosomes are show in Figure 1 but not discussed here.

After DNA replication, each chromosome consists of two identical sister chromatids attached by cohesin molecules. It is generally believed that chromosomes are separate units, not connected to each other. The reason why interphase chromosomes are not visible is related to the impermeability of the cellular and double nuclear membranes [31]. The two faces of the double-layered nuclear membrane are connected through a nuclear pore. Nuclear complexes are formed during the interphase by the insertion into the intact nuclear envelope. Nuclear membrane proteins, the lamina and chromatin form a network of finely coordinated interactions [32].

Two general mechanisms exist that control the macromolecular exchanges between the nucleus and cytoplasm depending on the physical and chemical properties of the permeant molecules The modulation of these properties affects both diffusion and active transport across the nuclear envelope [33].

## 3. Methods Applied for Permeabilization

The cell theory stating that cell membranes exist was recognized only when osmosis and the permeability of membranes gained more recognition [34]. Ernest Overton proposed that cell membranes consist of lipids [35]. The definition of permeabilization in microbiology involves the treatment of cells with mild surfactants that dissolve cell membranes allowing larger molecules to enter the cells. The earlier definition of permeabilization states that permeabilization removes cellular membrane lipids and provides access to large molecules through the membrane. The fixation and permeabilization permit not only the detection of cellular structures but also provide information about newly characterized proteins [36]. Besides organic solvents applied for the fixation of the cells, surfactants were commonly used for permeabilization [37]. For the isolation of human leukocytes, whole blood fixation or permeabilization with red blood cell lysis was employed [38]. Intracellular product release was achieved by chemical permeabilization [39]. The methods applied for the permeabilization of biological membranes distinguished several procedures including physical methods (among them osmotic lysis such as hypotonic treatment); pore-forming proteins; organic solvents, among them alcohols (methanol, ethanol), acetone, ether, toluene; mild detergents; enzymatic digestion and cryoprotectants. An important aspect of permeabilization is the prevention of chromosomes from escaping the nucleus, such agents are affecting membrane fluidity (e.g., dextran) [39].

Higher-order folding and chromosome condensation methods in mammalian cells were described in the 1970s [40,41]. The existing models of chromosome condensation [42,43] were contradictory and hypothetical since there were no methods, which would have permitted manipulations with the dense chromatin material. Due to the technological limitations, methods to follow large-scale chromatin changes did not exist. To resolve this problem, we applied reversible permeabilization to maintain cell viability and replicative DNA synthesis [12]. Our work related to chromosome condensation started with permeabilization and DNA synthesis in permeable cells. Intact cells incorporate thymidine but are impermeable to the larger and polar nucleotides. On the contrary, permeabilized cells that were originally unable to incorporate thymidine can take up deoxyribonucleoside triphosphates. Permeable cells can perform repair DNA synthesis in the presence of dNTPs without the energy of ATP, and can synthesize DNA in a replicative manner in the presence of dNTPs and ATP, but are prone to lose their viability, explaining the reduction in the time of permeabilization and the prevention of disruption of the cells in a hypotonic medium. Permeable mammalian cells which were originally unable to incorporate the radioactive nucleosides during the reversal of permeabilization regained their capacity to utilize thymidine and maintained their viability [44].

To overcome the permeability barrier that prevents the opening of nuclei, we adapted reversible permeabilization. This allowed the visualization of new interphase chromosomal structures. It was detected that freshly prepared thymocytes continued to synthesize DNA under hypotonic conditions in the presence of 4.5% dextran T-150, the four deoxyribonucleoside triphosphates and ATP. Permeable cells could seal the membrane in a serum-enriched medium within a few hours [12].

Intact cells incorporate thymidine but are impermeable to the larger and polar nucleotides. On the contrary, permeabilized cells that were originally unable to incorporate thymidine can take up deoxyribonucleoside triphosphates. The incorporation of nucleosides and nucleotides into reversibly permeabilized cells can be utilized to study macromolecular biosynthetic processes (DNA, RNA, poly-ADP-ribose), visualization of intermediates of chromatin condensation, apoptotic chromatin changes upon treatment with different genotoxic agents [5].

Chemical and physical methods are used to deliver large molecules inside cells. The major types of chemicals used for the permeabilization of cells are organic solvents and detergents.

### 3.1. Physical Methods

These methods consist of electroporation, serving as an efficient gene delivery system [45], electrofusion [46], microinjection [47], transfection [48], liposome-mediated drug delivery [49], the fusion of cell ghost [50], cell-penetrating peptides to deliver therapeutic proteins and oligonucleotides [51], osmotic lysis [52] and laser pulses for the delivery of therapeutics into cells [53] or by glass beads [54]. As an example, mouse L cells grown in suspension culture could be rendered permeable to exogenous deoxynucleoside triphosphates by a cold shock in a slightly hypotonic buffer system containing 0.01 M Tris-HCl pH 7.8, 0.25 M sucrose, 1 mM EDTA, 30 mM 2-mercaptoethanol and 4 mM MgCl_2_ [6,7,8]. Attention is called to the osmotic treatment as our approach to visualizing interphase chromosomes is based on similar hypotonic treatment.

#### 3.1.1. Permeabilization by Pore Formation in Membranes

Pore-forming toxins, e.g., sticholysin [55], streptolysin O [56] and pore-forming proteins such as transmembrane proteins, cause pyroptosis, known as an inflammatory form of cell death [57]. ATP-induced pore-forming toxins (e.g., nigericin derived from *Streptomyces hygroscopicus* and channels such as lysenin [58] cause channelopathies [59]. Coelenterazine is the general substrate for marine luciferases. It rapidly permeabilizes the plasma membrane and emits near-infrared bioluminescence for the imaging of mammalian cells [60].

#### 3.1.2. Organic Solvents

Alcohols: methanol showed a cytotoxic and antiproliferative effect in MCF-7, HepG2 and B16F10 cell lines [61]. Ethanol exposure suppresses mitochondrial dynamics and sensitizes mitochondrial permeability transition pore opening to promote cell death [62]. Acetone permeabilization allowed bypassing the fixation process during the staining of actin filaments for the visualization of muscle fibers in *Caenorhabditis elegans* [63]. The permeabilization of bacterial cells is recommended by freezing them and then treating the thawed material with an organic solvent such as chloroform, toluene or diethyl ether [64]. For the study of 5′-ends of nascent DNA, we have permeabilized *B. subtilis* cells with toluene [5].

#### 3.1.3. Mild Detergents

Non-ionic detergents served to permeabilize plasma membrane [61,62,63]. The optimization of other commonly used permeabilizing agents included the use of different saponin preparations, digitonin and recombinant lysing solutions such as perfringolysin O [64]. The capabilities were demonstrated by monitoring cell responses to permeabilization with Triton X-100 [65]. For the assessment of different methods and minimizing damage to the adherent cells, a variety of detergents and enzymes including saponin, triton X-100, tween-20, NPA40, proteinase K and streptolysin were used to optimize the protocols of permeabilization [66]. DNA synthesis was performed in lysolecithin-treated permeable cells [67]. This protocol was applied in permeable CHO cells synchronized in the S phase [68]. Smeared chromatin suggested abandoning the use of mild detergents for the isolation of interphase chromosomes.

#### 3.1.4. Enzymatic Digestion

Incubation in the presence of detergent and proteinase K with cells permeabilized cells and disrupted viral particles [66]. We have used proteinase K exclusively to lyse cytoplasmic, nuclear and membrane proteins. 

### 3.2. Agents Affecting Membrane Fluidity

Among permeabilization methods there are protective and fluidifying agents. Organic solvents (dimethyl sulfoxide, dimethylformamide) induce mitochondrial swelling, enhance the permeation to H^+^/K^+^, collapse the potential of the inner mitochondrial membrane and increase its fluidity [69].

#### Cryoprotectants

These can be penetrating agents or impermeable cryoprotectants. Cryoprotective media with a combined composition of mono- and di-saccharides have been developed [70,71].

## 4. Methods

### 4.1. Reversible Permeabilization

Our work related to chromatin and chromosome condensation started with the permeabilization of bacteria. Toluene served as a permeabilizing agent for *B. subtilis* cells to study discontinuous DNA replication, analyze the 5′-ends of Okazaki fragments and determine the size and sequence of RNA primers in Okazaki fragments [5,10]. The 5′-end analysis of *B. subtilis* nascent DNA was extended to murine thymocytes [12]. Reversible permeabilization was adapted not only to the analysis of the nascent DNA but also to visualize chromatin structures in mammalian chromosomes, initially in CHO cells. The method of reversible permeabilization originally was developed for murine thymocytes [15] and then adapted to CHO cells [72]. Briefly, 1 mL Hypotonic buffer was added to 10^6^ cells in the presence of dextran-150 as a molecular coat to prevent cells from disruption. Permeabilization lasted for 15 min at 0 °C. The time of permeabilization was gradually reduced. Recently, we use and recommend 1 min for permeabilization. For the reversal of permeabilization, the hypotonic medium was replaced by 10 mL of F-12 medium containing 10% fetal bovine serum and the cells were incubated in a CO_2_ incubator at 37 °C for 3 h.

### 4.2. Cells

#### 4.2.1. Murine Thymic Cells

Thymus cells were freshly isolated from male CFLP mice and immediately used for permeabilization [12].

#### 4.2.2. CHO-K1

The chromatin structure of the Chinese hamster ovary (CHO-K1 ATCC CCL61) hypodiploid, aneuploid cell line [72], ranging between 18 and 42 chromosome numbers was investigated. The frequency of distribution of chromosomes in these cells was 2n = 22 and the modal chromosome number was 20.

#### 4.2.3. Indian Muntjac (IM) Cells

The *Indian muntjac* cell line was obtained from cells by skin biopsy from an adult muntjac deer. This cell line is advantageous to study chromosomes due to the low chromosome number [73,74]. The aneuploid Indian muntjac male cell line (2n  =  9, with trisomy 1) was obtained from Karl Sperling [75]. *Muntiacus muntjak* cells were grown in suspension culture at 37 °C in RPMI 1640 medium containing 5% fetal bovine serum. Chromatin structures of exponentially growing interphase cells were visualized after reversible permeabilization and staining of DNA with the fluorescent stain 4′,6-diamidino-2-phenylindole (DAPI). The spatial arrangement of Indian muntjac chromosomes was visualized and their linear attachment and order were determined.

*Drosophila Schneider 2* cells. This cell line was obtained from B.Z. Shilo. The structure of *Drosophila* chromosomes in nuclei was described earlier in *Drosophila Schneider 2* cells [76].

#### 4.2.4. Myeloid l—Leukemia Cells

The myeloid leukemia cell line (MyDe I) was established [77,78] from myelomonocytic leukemia cells that were implanted under the renal capsule of F344, Long–Evans and BDIX rats [79]. Exponentially growing human erythroleukemia K562 cells [80] were used for permeabilization, subjected to reversible permeabilization and isolation of interphase chromosomes [44]. 

The characteristic elongated and linearly attached chromosomes in myelogenous leukaemia cell line with positive Philadelphia chromosome was discovered in 1975 [80]. Methotrexate-induced apoptotic and necrotic chromatin changes were seen in rat myeloid leukemia cells [81].

### 4.3. Nascent DNA Synthesized in Reversibly Permeabilized Cells

**Figure 2:**
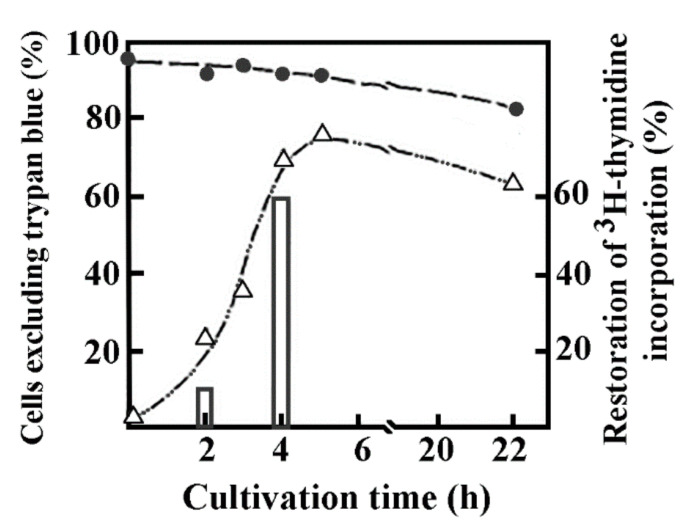
Regeneration of permeabilization tested by trypan blue staining and 3H-thymidine incorporation in murine thymocytes. Mouse thymus cells were permeabilized and then diluted with Parker-199 medium enriched with 10 % human AB serum. Sealing of cells after permeabilization was secured by the presence of dextran T-150 in the medium. The viable cells were measured by the trypan blue exclusion test and expressed as percentages of the total cells (∆). Simultaneously, the viability of intact cells (●), and the [3H]thymidine incorporation (∆) after permeabilization were followed and expressed as percentages of [3H]thymidine incorporation of intact cells. Reprinted with permission from Ref. [12]. Copyright year 1984, Eur. J Biochem Figure 2 in this ref.

DNA synthesis in reversibly permeabilized murine thymic cells followed the strategy applied to study early intermediates of nascent DNA in bacterial cells. Briefly, the modified version of Halldorsson et al. [82] was applied to mouse thymus lymphocytes in a hypotonic solution. The hypotonic solution consisted of 9 mM HEPES pH 7.8, 5 mM dithiothreitol, 4.5% dextran (*w*/*v*) 150,000 mol. wt, 1 mM EGTA, 4.5 mM MgCl_2_. Not only lymphocytes but cells from different sources (murine spleen, human tonsillar lymphocytes, Indian muntjac, rat myeloid leukemia, murine pre-B, *Drosophila*) could be permeabilized up to 5.5 × 10^7^ cells/mL. About 95% of murine thymic cells became permeable rapidly. The time of permeabilization was reduced first from 15 to 2 min then from 2 min to 1 min in myeloid leukemia cells at 0 °C. The permeabilization was followed by the regeneration of cells by replacing the hypotonic solution with a bovine serum enriched (10%) medium resulting in the restoration of the integrity of the cell membrane in the majority of cells measured by trypan blue dye exclusion. The kinetics of cell regeneration was measured by the incorporation of [^3^H]-thymidine characteristic only to intact cells, whereas permeable cells were unable to utilize this nucleoside for DNA synthesis but rather could efficiently incorporate nucleoside triphosphates in an ATP-dependent manner. Permeable cells have temporarily lost their ability to incorporate [^3^H]-thymidine, but ~60% of permeabilized cells regained their capacity to incorporate the radionucleotide after a 4 h regeneration period. The loss of viability of control cells where no permeabilization occurred was 5%, in permeable cells it was approximately 10% after an extended 22 h incubation (Figure 2). Scanning electron microscopy confirmed the regeneration of cells.

Figure 2 shows the morphology of intact, permeable and sealed cultures of murine thymocytes. Control and sealed cells are similar, but still distinguishable by the somewhat larger size and scarred appearance of regenerated cells. Drastic changes in the surface structure of permeabilized cells are indicated by their oval shape, tendency to stick together and hollows indicating the loss of membrane integrity.

Different methods not only aim to make cells permeable but also maintain the functional integrity of the cells. The multitude of methods indicates the lack of a unique, fast and reliable solution to permeabilization that could be of general use. Chemical agents that disrupt the nuclear membrane release nucleic acids resulting in a sticky mass of the nuclear material requiring additional protective measures to prevent the disruption of nuclei. The advantages and disadvantages of permeabilization methods have been reviewed [79].

**Figure 3:**
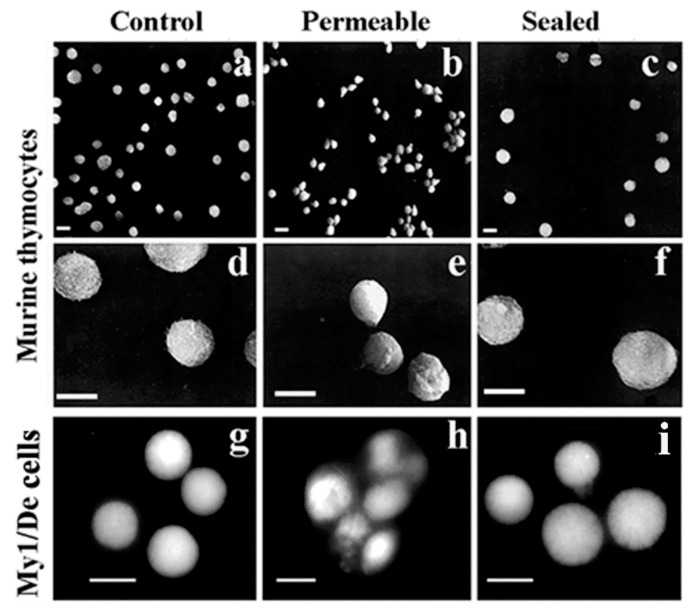
Reversible permeabilization of murine thymocytes and myeloid leukemia (My/De) cells visualized by scanning electron microscopy. (**a**) Control thymocytes were not permeabilized. (**b**) Permeabilized thymocytes, (**c**) sealed thymocytes permeabilized for 15 min kept in the presence of 4.5 g/v-T150 and hypotonic buffer. (**c**) Sealed in the presence of a K10 medium containing 10% fetal bovine serum. Thymocytes at higher magnification: (**d**) Control cells, (**e**) permeable thymocytes, (**f**) after reversible permeabilization. My1/De cells: (**g**) control, (**h**) after 1 min permeabilization, sealed cells after permeabilization (**i**). Scale bar 5 µm. Modified with permission of Ref. [12]. Copyright year 1984, Eur. J. Biochem Figure 2.

After testing several methods to open the nuclear membrane, and trying to keep nucleic acids inside the nucleus, the modified protocol of the growth medium of the Halldorsson group [82] contained 10% fetal bovine serum selected, in a slightly hypotonic buffer. The presence of dextran T-150 served as a molecular coat to prevent murine thymocítes cells from premature disruption (Figure 3a–f). The regeneration of permeable cells took place in a growth medium containing10% fetal bovine serum. The applicability of reversal of permeabilization is demonstrated in MyDe l cells including control (Figure 3g), permeabilized cells generating a sticky mass (Figure 3h) and sealed cells after reversal of permeabilization (Figure 3i).

#### Growth of Cells

*Murine thymocytes.* Freshly isolated cells were resuspended in Eagle’s medium and stored at 4 °C at 5 × 10^6^ cells/mL for not longer than 1–2 h until further use. Cells were grown in Eagle’s medium. The viability of cells was above 95% as determined by trypan blue dye exclusion [12].

*CHO-K1*. With a low chromosome number (2n = 22) the mammalian Chinese hamster is a good model for cytogenetical and tissue culture experiments [72]. Initially, CHO K1 (ATCC #CCL61) were grown either in suspension culture in spinner flasks using minimal Eagle’s medium (MEM) plus 0.3 mM L-proline or in plastic 75 cm^2^ flasks using F12 Ham’s medium both supplemented with 10% fetal bovine serum (FBS) in a carbon dioxide incubator at 37 °C [73]. For large-scale growth, CHO cells (10^6^) were grown in a K10 medium containing 5% fetal bovine serum. The growth of the cell culture after 12 h of inoculation was seen by the elongation of originally round cells. Confluency of the monolayer was attained after 72 h.

*Indian muntjac (IM)* cells. The *Indian muntjac* cell line was obtained from cells by skin biopsy from an adult muntjac deer. This cell line is advantageous to study chromosomes due to the low chromosome number [73,74]. Karl Sperling obtained the aneuploid Indian muntjac male cell line (2n = 9, with trisomy 1) [75]. Cells were grown in PMI-1640 medium supplemented with 10% fetal bovine serum.

*Drosophila Schneider 2* cells. This cell line was described earlier [75]. Cells were cultured in *Drosophila Schneider 2* cell [76]. Cells were grown in suspension culture at 25 °C using Schneider’s medium supplemented with 10% FBS. 

*Myeloid leukemia* and human *erythroleukemia* cells were grown in PMI-1640 medium supplemented with 10% fetal bovine serum.

### 4.4. Reversible Permeabilizations of Cells

For the permeabilization of murine thymocytes, we have used a slightly hypotonic buffer containing dextran T-150 [12] and have adapted this system to study DNA replication in different cell types. The time of permeabilization was reduced to 1 min at 0 °C. 

#### 4.4.1. Isolation of Nuclei from Permeabilized Cells 

CHO-K1 cells (2 × 10^6^) were grown in a K-10 medium containing 5% fetal bovine serum and during the reversal of permeabilization in a medium containing 10% fetal bovine serum and kept at 37 °C for 3 h then treated with 0.1 µg/mL colcemid. After further incubation for 2 h at 37 °C cells were detached and washed with PBS, kept at 37 °C for 10 min in 10 mL of 50 mM MgSO_4_, 3 mM dithiothreitol and 5 mM Na_2_HPO_4_/NaH_2_PO_4_ pH 8.0 swelling buffer. Nuclei were centrifuged for 8 min at 500× *g* and fixed in 10 mL methanol: glacial acid (3:1) fixative. It deserves mention that reversible permeabilization has an advantageous swelling effect on cells seen by their slight enlargement (Figure 3c,f,i). 

#### 4.4.2. Visualization of Interphase Chromatin Structures and Chromosomes

Nuclei isolated from permeabilized cells were prepared the same way as those of control cells. Nuclei were centrifuged at 500× *g* for 8 min washed twice with 1 mL fixative, and spread over glass slides dropwise from a height of about 30 cm. Slides were air-dried, stored at room temperature overnight, rinsed with PBS and dehydrated using increasing concentrations of ethanol from 70 to 100%. Dehydrated slides were air-dried and before microscopy mounted in 35 µL antifade medium under 24 × 50 mm coverslips. Blue fluorescence of DAPI stained (25 ng/mL) DNA was monitored under a Dialux 20 fluorescent microscope (Leitz).

## 5. The Linear Arrangement of Intermediates of Chromatin Condensation

### 5.1. Intermediates of Chromatin Folding in Nuclei of CHO-K1 Cells

**Figure 4:**
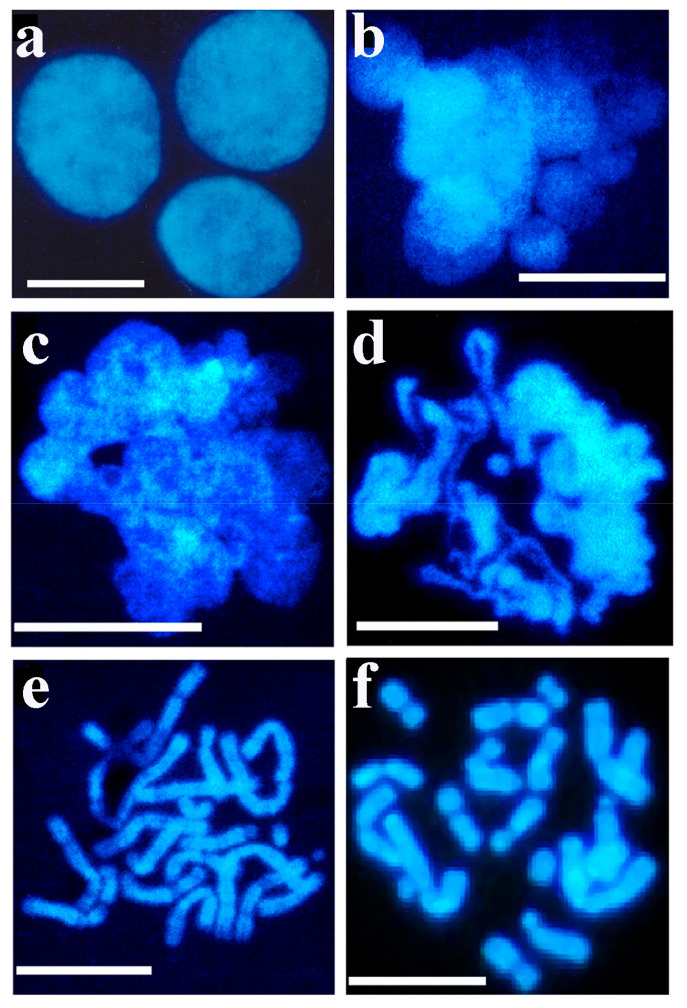
Isolation of interphase and metaphase chromosomes from exponentially growing CHO cells. A 30 min pulse-label of nascent DNA in permeable cells in the presence of the four dNTPs was followed by the reversal of permeabilization. Cells were kept at 37 °C for 3 h and then treated with 0.1 µg/mL Colcemid. After further incubation (2 h at 37 °C), cells were detached with trypsin and washed with PBS. Swollen cells were centrifuged at room temperature (800× *g* for 5 min), and fixed in 10 mL methanol: glacial acid (3:1). Preparation of spreads of metaphase chromosomes was the same as that of interphase nuclei. The reversible permeabilization of cells, isolation and visualization of chromatin and chromosome structures were described in the Methods. (**a**) Control nuclei isolated from reversible permeable cells, (**b**) metaphase chromosomes, (**c**) nuclear ribbon-forming loops, (**d**) chromatin loops turning to chromosomes, (**e**) bent, elongated chromosomes and (**f**) metaphase chromosomes. Bars, 5 µm each. Modified with permission from Ref. [84]. Copyright year 2014, Apoptosis in Figure 4 of this reference.

Permeabilized CHO-K1 cells were incubated in a growth medium containing newborn bovine serum (10%). It was found that the regeneration of the cellular membrane gradually slowed down. After 4 h of incubation in the presence of newborn serum approximately 50% of cells regained their ability to incorporate [^3^H]-thymidine and only 22% in the presence of low serum concentration. The kinetics of cell regeneration showed that this process slowed down within a few hours. In the control experiment without permeabilization, about 5% of the cells were damaged as a result of experimental manipulation. Pulse-chase experiments were carried out but failed to drive biotinylated DNA from interphase to metaphase, rather the separation and expulsion of biotinylated DNA were observed [83]. An early indication of the presence of decondensed chromatin in permeable interphase nuclei (Figure 4a) after the nucleus opened, was the appearance of chromatin spheres (early chromosomal forms) (Figure 4b), chromatin loops (Figure 4c), ribbons of chromatin turning to chromosomes (Figure 4d), early bent and elongated chromosomes (Figure 4e) and chromosomes approaching metaphase (Figure 4f).

### 5.2. Isolation of Interphase Chromosomes from Synchronized Cells

**Figure 5:**
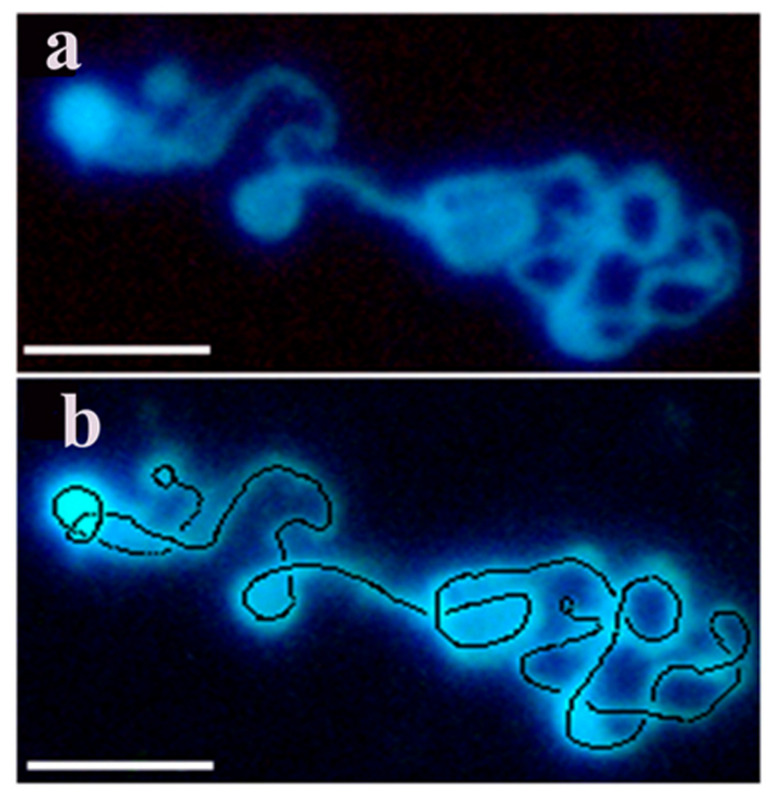
The continuous backbone of the decondensed chromosome of CHO-K1 is traced with a dotted black line and its two ends are indicated by small back circles. Decondensed chromatin fiber was isolated from reversibly permeabilized CHO cells. (**a**) Isolation and visualization of the linear array of chromatin were performed as described in the Methods. (**b**) The same c chromatin structure is indicated by the continuous line and the two ends of the nuclear material. Bars, 5 µm each. Modified with permission from Ref. [30]. Copyright year 2009, Springer, Dordrecht. Figure 3.14 of this ref.

From intact cells only interphase nuclei and metaphase chromosomes could be isolated (see at Figure 1). To overcome the technical hindrance chromatin structures were isolated from nuclei after a short pulse of permeabilzation in a slightly hypotonic solution where the danger of loosing cellular components was compensated by dextran as a molecular coat in the permeabilizing solution, to maintain viability, yet able to open nuclei without inducing apoptosis. This powerful method allowed to monitor the kinetics of chromatin condensation in a time-lapse manner during the cell cycle using synchronized cells. By using this new approach we have confirmed the expected flexible folding pattern by visualizing transient characteristic forms of condensing interphase chromosomes. As model systems we have isolated murine lymphocytes, CHO-K1 cells, murine pre-B, rat hepatocarcinoma, human erythroleukemia, Drosophila, Indian muntjac, garden snail chromosomes. Most of the experiments were performed with CHO-K1 cells. Figure 5 provides evidence that decondensed chromain string is a continuous structure with loops forming chromosomes.

#### 5.2.1. Chromatin Structures from Decondensed Chromatin to Metaphase Chromosomes

**Figure 6:**
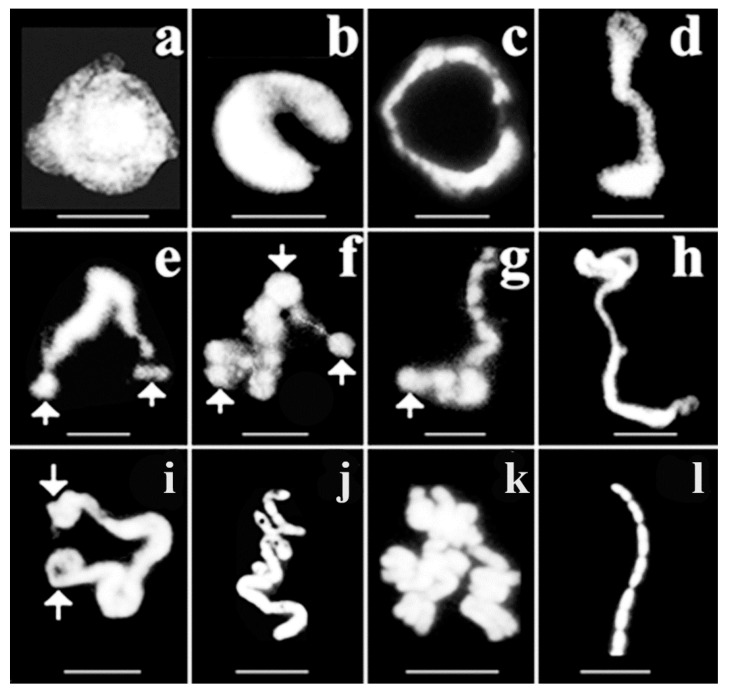
Linear connection of interphase chromosomes in condensing chromatin. Chinese hamster ovary cells were kept in suspension culture in spinner flasks. Exponentially growing cells were synchronized by elutriation and eight fractions were collected. Details of cell growth, synchronization, FACS analysis, reversal of permeabilization, isolation of nuclei, spreads of nuclear structures and visualization of chromatin structures were carried out. CHO chromosomes are synchronized by centrifugal elutriation representing different stages of the cell cycle. Condensing chromosomes were selected from the multitude of forms that were characteristic of the cell cycle they belonged to. Reversible permeabilization, isolation of nuclei, spreads of nuclear structures and immunofluorescent visualization of interphase and metaphase chromosomes were described in [83]. (**a**–**d**) Opening CHO-K1 nuclei forming semicircular linear chromatin. (**e**–**g**). Formation of interphase chromosomes indicating the clustering inside the chromatin. (**h**,**i**) chromatin ribbon turning to zig-zag arrangement of elongated chromosomes. (**j**,**k**) Pre-condensed chromosomes. (**l**) Linearly connected metaphase chromosomes. Bars, 5 µm each. Modified with permission from Ref. [30]. Copyright year 2009, Springer, Dordrecht. Figure 3.17 of this ref.

Large-scale chromatin changes are shown here which take place in the interphase nucleus. Synchronized CHO cells were permeabilized reversibly and used to isolate chromatin structures. Figure 6 visualizes changes which reflect connectivity both in decondensed chromatin and in condensing chromosomal structures. Synchronized CHO cells were permeabilized reversibly and used for the isolation of chromatin structures. 

In the early S phase chromatin appeared as a helical structure (“cartwheel” pattern). The nuclear material maintained its round shape with a polarized end-plate emerging from the nucleus (Figure 6a) that gradually became came unfolded (Figure 6b). The extruded veiled chromatin then developed into supercoiled loops (Figure 6c). Further coiling formed a continuous ribbon structure (Figure 6d). Supercoiling generated chromatin bodies (Figure 6e–g). Further coiling turned the whole chromatin structure into round shaped bodies (Figure 6f). These supercoiled bodies are regarded as the earliest visible forms of chromosomes in the interphase nucleus. The next level of condensation leads first to elongated chromatin bodies (Figure 6g), then to thick chromatin fibers (Figure 6h). Arrows in Figure 6e–f indicate the formation of chromatin bodies. Further evidence for a continuous, linear chromatin structure is provided by Figure 6i, where thinner chromatin fiber is converted to a relatively loose but thick chromatin rope. Supercoiling tightens the rope and generates loops, which correspond to elongated prechromosomes arranged in a regular three dimensional zig-zag array (Figure 6h,i). This structural arrangement should not be confused with a lower level of folding, namely the so called 30 nm chromatin fiber, where there is a possibility that nucleosomes can be organized in a three-dimensional zig-zag according to the availability of space and numbers of chromosomes. At the end of the folding process linearity seems to be maintained in folded prechromosomes (Figure 6j,k). Linear connection is clearly maintained in metaphase chromosomes (Figure 6l). 

#### 5.2.2. Chromatin Image Analysis of Condensed Chromosomes inside the Nucleus of CHO Cells

**Figure 7:**
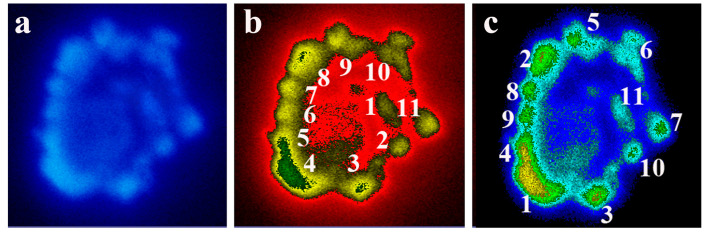
Semi-circular (not closed) arrangement of pre-chromosomes in nuclei of CHO K1 cells synchronized at the end of S-phase (3.5–4.0 C). CHO cells at the end of S phase stages in S phase were obtained by centrifugal elutriation. Chromatin Image Analysis of condensing chromosomes. (**a**) The semicircular arrangement of chromosomes in a CHO-K1 nucleus. The linear arrangement of chromosomes seen after the DAPI staining of nuclear material. (**b**) Chromatin image analysis: Numbering of linearly arranged chromosomes starting at the chromosome closest to the largest (number 1) chromosome. (**c**) Numbering based on chromosome size. Size of nucleus, 5 µm each. Modified with permission from Ref. [30]. Copyright year 2009, Springer, Dordrecht. Figure 3.16 of this reference.

Interphase chromosomal structures showed a variety of transition forms from decondensed to gradually condensing chromosomes that have been analyzed by computer programs. Briefly, for 3D visualization of chromatin images, fluorescent light was detected by a CCD camera attached to the microscope. The image was digitally stored with red, green and blue tonality of pixels. Our software termed ‘Chromatin Image Analysis’ (CIA) resulted in characteristic data on each pixel: X and Y coordinates, as well as the monochromatic intensity of the pixel. In the next step, the program separated the valuable information and catted out the noise from the image. The user can make different processing steps to optimize the database, e.g., fit precalculated intensity to the image, add colored masks to the 3D objects, objects independently displayed from the software and plugins, simultaneously attached with the microscopic procedures in a real-time dependent manner. The software was developed in 2001 [83] and required Win9x, Win2000, NTOS’ and DirectX 8.0 extensions. The separation of valuable information and the removal of noise improved the quality of the images. The chain consisted of 11 chromosome, not much longer than 10 chromosomes and the chromosome order could be determined. Steps of chromatin image analysis are shown in Figure 7.

### 5.3. Chromosome Order in Drosophila Cells

Although, the correlation between the number of subphases of DNA replication and the number of chromosomes is not completely understood, the linear arrangement of chromosomes suggests that chromosome replication is related to a defined temporal order of chromosome condensation. Similarly, to CHO cells the linear arrangement of chromosomes was demonstrated in *Drosophila*, Indian muntjac cells. Moreover, the sequence of chromosomes can be determined in a few cells where the number is not high. In cells with higher chromosome numbers (e.g., in human cells n = 23), chromosomes jump over each other making the judgment of chromosome order impossible.

**Figure 8:**
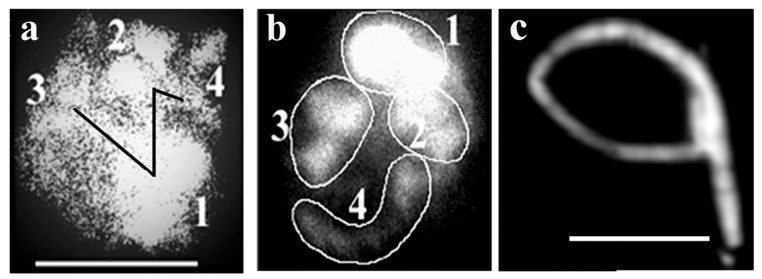
Determination of linear order of chromosome attachment in Drosophila nuclei. Chromosomes are numbered based on their size. Two sets of interphase chromosomes representing different stages of condensation are seen in panels (**a**,**b**). Pairs of chromosomes are circled in (**b**). Condensed Drosophila nuclear material without well distinguishable chromosomes (**c**). Bar 5 µm, each. Modified with permission from Ref. [30]. Copyright year 2009, Springer, Dordrecht. Figure 3.38 of this reference.

The *Drosophila* chromosome sequence is deduced from the fluorescence intensities. The characteristic butterfly shapes of chromosome pairs are in anaphase connected via chromatin bridges (black zig-zag line) and sister chromatids are about to separate to daughter chromosomes. Based on the fluorescence intensity and size, the largest chromosome (#1) is followed by numbers of decreasing size and fluorescent intensity of the rest of the chromosomes. Numbering starts at the chromosome that is closest to the largest (#1) chromosome. Figure 8b shows the circled pairs of homologous chromosomes. The sequence of *Drosophila* chromosomes based on elevating light intensities seen in Figure 8a,b is 3, 1, 2 and 4. Figure 8c contains linearly attached chromosomes approaching metaphase with individual chromosomes not distinguishable. 

### 5.4. Visualizing the Chromosome Order of Indian Muntjac Cells

**Figure 9:**
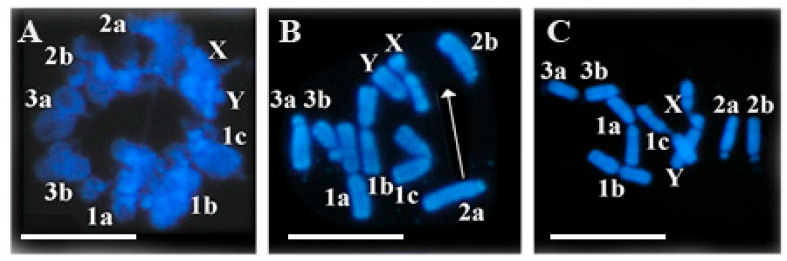
Linear attachment of Indian muntjac chromosomes. Diploid state is indicated for chromosomes 2 and 3 with letters (2a, 2b; 3a, 3b), chromosome 1 is triploid (1a, 1b, 1c), sex chromosomes are X and Y. (**A**) Interphase chromosomes. (**B**) Metaphase chromosomes with chromosome #2a abandoning its place during isolation (indicated by the white arrow), (**C**) properly ordered chromosomes and the sequence of metaphase chromosomes: 3,3, 1,1,1, y,x, 2,2. Scale bar 20 µm each.

Indian muntjac (*Muntiacus muntjak*) cells were grown in suspension culture at 37 °C in RPMI 1640 medium containing 5% fetal bovine serum. Chromatin structures of exponentially growing interphase cells were visualized after reversible permeabilization and staining of DNA with the fluorescent stain 4′,6-diamidino-2-phenylindole (DAPI). The spatial arrangement of Indian muntjac chromosomes was visualized and their linear attachment and order have been determined. When nuclei are spread over glass slides the splashing of metaphase chromosomes during the spreading of nuclear structures is likely to disrupt the continuity of the chromosomes. Some of the metaphase chromosomes may overlap each other or be distributed in a non-linear arrangement. Nevertheless, most of the chromosomes maintain their linear order and from the metaphase spread the linearly order of chromosomes can be determined. Figure 9 shows the non-random distribution of linearly attached inter- and metaphase chromosomes.

### 5.5. Interphase Chromosomes in Murine Pre-B Cells

**Figure 10:**
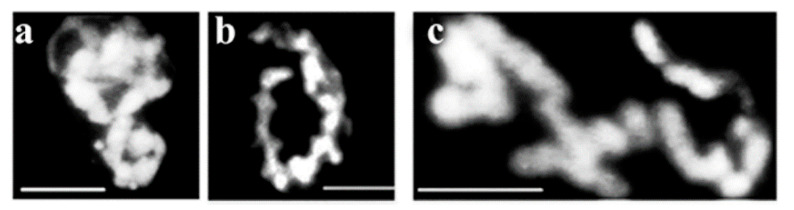
Chromosomes extruded from linearly arranged interphase chromatin. The murine pre-B cell line 70Z = 3-M8 was grown in suspension culture at 37 °C in RPMI 1640 medium supplemented with 10% fetal bovine serum (FBS), 2 mg = mL mycophenolic acid, 150 mg = mL xanthine, 15 mg = mL hypoxanthine and 2 × 10−5 M ß-mercaptoethanol. Permeabilization of cells (106) was followed by the restoration of membrane structures, isolation and visualization of chromatin structures, as described in the Methods. Panels (**a**,**b**) represent structures of linear connectivity between supercoiled chromatin loops, and (**c**) condense early chromosomes. Scale bar 5 µm each. Modified with permission from Ref. [30]. Copyright year 2009, Springer, Dordrecht. Figure 3.19 of this reference. Only examples of linearly arranged chromatin structures are shown in Figure 10. Besides the regular structures seen among the condensing chromosomes, other forms such as fibrous, looped chromatin are visible but not shown here.

We have studied genotoxicity specific stress in a murine pre-B cell line70Z/3 cells line [85] and show here the somewhat modified, nertheless linear attachment of condensing chromatin chromatin and chromosomes. Figure 10 shows linearly attached murine pre-B chromosomes. 

### 5.6. Organization Principles in Garden Snail (Helix pomata) Chromosomes

**Figure 11:**
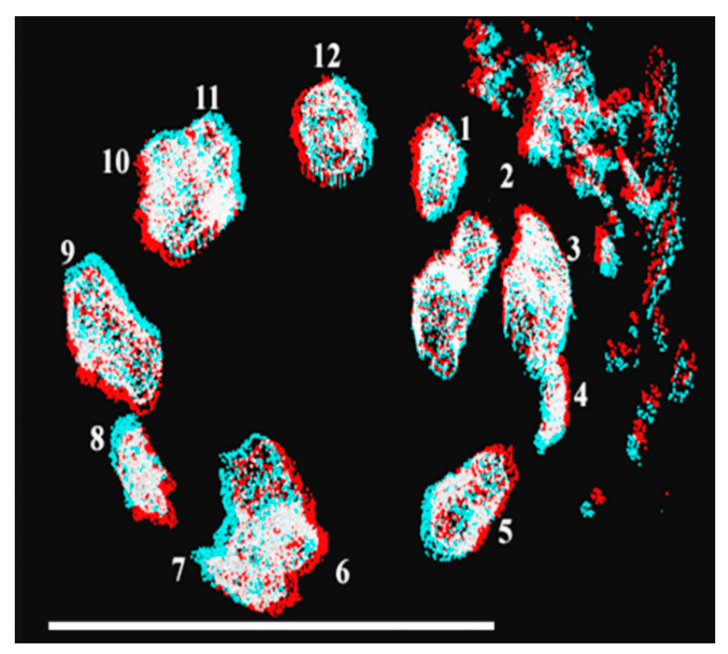
Anaglyph-transmission electron microscopy visualization of garden snail (*Helix pomata*) chromosomes. Numbered (1–12) haploid chromosomes of type B spermatogonia. The stereoscopic 3D effect is achieved using encoding each eye’s image using filters of different colors, typically red and cyan. TESLA BS 540 transmission electron microscope was used in digital processing mode (TEM). Bar, 20 µm. Modified with permission from Ref. [84]. Copyright year 2014, Apoptosis in Figure 4 of this reference.

The garden snails, such as *Helix aspersa* and *Helix pomata,* are among the best-known species in the world and the most proliferative terrestrial mollusks. These species dwell in several places also as agricultural pests feeding often on crops for food. It is easy to maintain and use them as model animals suitable to provide chemical compounds and develop new medicines for humans. Salamanders and snails are known to have 24 chromosomes, but the fact is that we do not know their exact chromosome number. We have isolated chromosomes from the garden snail *Helix pomata* and subjected them to 3D anaglyph electron microscopy (Figure 11).

The isolation and visualization of chromosomes of garden snails confirmed that the haploid chromosome number is 12. The chromosomes are lined up circularly and their compaction is low deduced from their relatively large size. The stuck-together chromosomes (3 + 4, 6 + 7, 10 + 11) suggest that chromosomes could have formed a continuous linear arrangement. However, this assumption needs confirmation.

## 6. Discussion

Cell membranes are semi-permeable allowing them to control those molecules that can enter or leave. As general rule water, small molecules and molecules without a charge can pass freely, for large molecules and ionic substances the cell membrane is impermeable. Eukaryotic cells, unlike prokaryotes, possess additional internal membranes that wrap these organelles to control the exchange of essential cell components. The best protection is provided to the nucleus that encloses the nuclear material with a double-layered membrane that permits the passage of certain molecules (nucleic acids and proteins) through the poles between the nucleus and cytoplasm.

### Utilization of Permeable Cells

The multitude of methods for different purposes indicates the importance and the interest in permeabilization. High levels of DNA synthesis in permeable cells were of the replicative type and replicon size [6,7,8]. Experiments related to the in vitro DNA replication of chromatin reflected the complete replication of SV40 DNA and chromatin [83]. Despite the many applications of permeabilization, chromatin condensation was not among them. Attempts were made to apply in situ hybridization techniques to explore chromosome and gene arrangement, but these techniques require conditions that denature DNA. The harsh conditions are antagonistic to examining specific chromosomal regions, higher-order chromatin structures that belonged to the scope of electron microscopy [84,85] and genomic techniques [86].

The fact that different condensing chromatin forms were not seen in interphase can be explained by the lack of methods for the cellular membrane. Metaphase chromosomes were visualized using methanol-acetic acid fixative and the standard fixation method. Only two types of nuclear structures became visible: the nucleus itself, and the metaphase chromosomes, as the cellular membranes, prevented the look behind the nuclear curtain. To break down the permeability barrier that prevent nuclei opening, we have adopted a mild treatment based on the reversible permeabilization that allowed the visualization of chromosomal structures throughout the cell cycle [85]. The argument that methanol-acetic fixation after permeabilization is damaging the architecture of chromatin structures by dehydration [87,88] is doubted since metaphase chromosomes have been isolated by the same procedure as other intermediates of chromatin condensation. To maintain the viability of cells and reduce the risk of chromatin damage during reversible permeabilization, we shortened the pulse of slightly hypotonic buffer treatment in the presence of T-150 and higher and higher fetal serum concentration (10%), during the healing of the cells which not only prevented the disruption of cells but also contributed to the healing of the cells. In agreement with our observations, experiments of others confirmed that an even more drastic and longer hypotonic treatment did not impact cell viability [89].

As linear connection of chromosomes was found in primary cell cultures (murine T cells), in other mammalian (Indian muntjac, CHO, murine pre-B and human K562 erythroleukemia) cell lines and lower eukaryotic (*Drosophila melanogaster, Helix lucorum*) cells, it is concluded that it is a general phenomenon that chromosomes attach throughout the cell cycle rather than being individual units. It did not escape the attention of the author that the chromosomes of the connection contribute to a better understanding of the Rabl model to adopt a stable territorial organization with far-reaching consequences to the chromosomal structure and function.

Permeable cells were not or were not used successfully by others to visualize interphase chromosomes. This is the reason why the author makes references primarily to their work but would like to see conclusions reinforced by others.

## Data Availability

Not applicable.
